# Label-Free Cross-Priming Amplification Coupled With Endonuclease Restriction and Nanoparticles-Based Biosensor for Simultaneous Detection of Nucleic Acids and Prevention of Carryover Contamination

**DOI:** 10.3389/fchem.2019.00322

**Published:** 2019-05-08

**Authors:** Yi Wang, Lin Sun, Jie-qiong Li, Ze-ming Wang, Wei-wei Jiao, Jing Xiao, Chen Shen, Fang Xu, Hui Qi, Yong-hong Wang, Ya-jie Guo, A-dong Shen

**Affiliations:** Key Laboratory of Major Diseases in Children, Ministry of Education, National Key Discipline of Pediatrics (Capital Medial University), National Clinical Research Center for Respiratory Diseases, Beijing Key Laboratory of Pediatric Respiratory Infection Disease, Beijing Pediatric Research Institute, Beijing Children's Hospital, Capital Medical University, National Center for Children's Health, Beijing, China

**Keywords:** cross-priming amplification, endonuclease restriction, lateral flow biosensor, *S. aureus*, limit of detection

## Abstract

Here, we reported on a label-free cross-priming amplification (CPA) scheme that utilized endonuclease restriction for simultaneous detection of nucleic acids and elimination of carryover contamination. Reaction mixtures were detected in a nanoparticle-based lateral flow biosensor (LFB). The assay exhibited attractive traits in that it did not require the use of labeled primers or labeled probes, and thus, the technique could prevent undesired results arising from unwanted hybridization between labeled primers or between a probe and labeled primer. Isothermal amplification and endonuclease restriction digestion were conducted in a single pot, and the use of a closed-tube amplification removed false-positive results due to contaminants. To validate the assay's applicability, we employed the novel technique to detect the pathogen *Staphylococcus aureus* in pure cultures and artificial blood samples. The assay could detect target bacterium in pure culture with a 100 fg.μL^−1^ detection limit, and in spiked blood samples with a 700 cfu.mL^−1^ detection limit. The whole process, including sample procedure (20-min), isothermal amplification (60-min), endonuclease digestion (10-min) and result reporting (within 2-min), could be finished within 95-min. As a poof-of-concept assay, the technique devised in the current report could be employed for detecting various other sequences if the specific CPA primers were available.

## Introduction

Nucleic acids were widely used not only for biological research but also as powerful biomarkers for forensic science, agriculture, clinical diagnosis, environmental monitoring, food safety test, and so on. Polymerase chain reaction (PCR)-based techniques, as the well-known molecular tools, have been applied for amplifying and analyzing low-abundance nucleic acids (Niemz et al., 2011; Feng et al., 2018a). However, these technologies require complex apparatus for amplification temperature adjustment and trained personnel, which significantly hinders their application in resource-limited settings (Reid et al., 2018). Additionally, these methodologies have mispriming and sometimes inadequate template amplification. Herein, device of inexpensive and simple techniques for nucleic acids amplification and detection is extremely important for on-site diagnostic applications in place of PCR-based assays.

To this end, extensive numbers of isothermal amplification technologies, including strand displacement amplification (SDA), helicase-dependent amplification (HDA), recombinase polymerase amplification (RPA), multiple cross displacement amplification (MCDA), loop-mediated isothermal amplification (LAMP) and cross-priming amplification (CPA), have been proposed (Wang et al., 2015, 2018a; Zhao et al., 2015; Wachiralurpan et al., 2018). Among these techniques devised for detection and analysis of target sequences, CPA was a powerful innovative amplification method, and has been widely used in a variety of areas (Liu et al., 2018; Meng et al., 2018). CPA assay was able to achieve the amplification of nucleic-acid sequence at a constant temperature, and only an enzyme with strand displacement activity and a set of five primers were required for conducting the CPA reaction (Xu et al., 2012). Usually, CPA result was indicated by costly fluorescence-based techniques, by tedious gel electrophoresis, or by the used of spectrophotometric apparatus to measure turbidity. Either complicated and bulky optical apparatus or exposure to carcinogen dyes was required, thus precluding their wider application in laboratories with limited resources settings (Wang et al., 2016). In addition, colorimetric indicators, such as pH-sensitive dyes (neutral red), malachite green (MG), and hydroxynaphthol blue (HNB), were also employed as the complementary techniques for monitoring CPA reactions (Xu et al., 2012). Nevertheless, a tested sample, which contains the extremely low concentration of target nucleic acid templates, may be equivocal, because the observation of color with naked eye is potentially subjective. As such, the newer monitoring techniques, which are capable of simplifying and speeding up the total time of CPA-based methods, should be devised.

More recently, nanoparticles-based lateral flow biosensor (LFB), which has become a promising stool for analyze various analytes (Cai et al., 2018; Duan et al., 2018), has been employed as an alternative technique for reporting CPA results (Huo et al., 2017; Gou et al., 2018). In order to facilitate biosensor detection of CPA products, two primers (i.e., 2s/3s) involved in CPA reaction are simultaneously modified, one with hapten (i.e., FITC, Dig, and Hex) and one with biotin (Meng et al., 2018). During the amplification stage, both 5′- end of the amplified products are simultaneously modified with hapten and biotin. The labeled CPA products (Biotin-dsDNA-hapten) can be determined by nanoparticles-based biosensor, and the amplification results are indicated as a colored band by naked eye within 5 min. Unfortunately, the pivotal disadvantage of the traditional strategies is that unwanted results can be obtained from undesired interaction between two modified CPA primers, because a double-modified detectable product can be formed by the undesired hybridization from the two labeled primers (one with biotin, and one with hapten) without CPA amplification.

**Figure 1:**
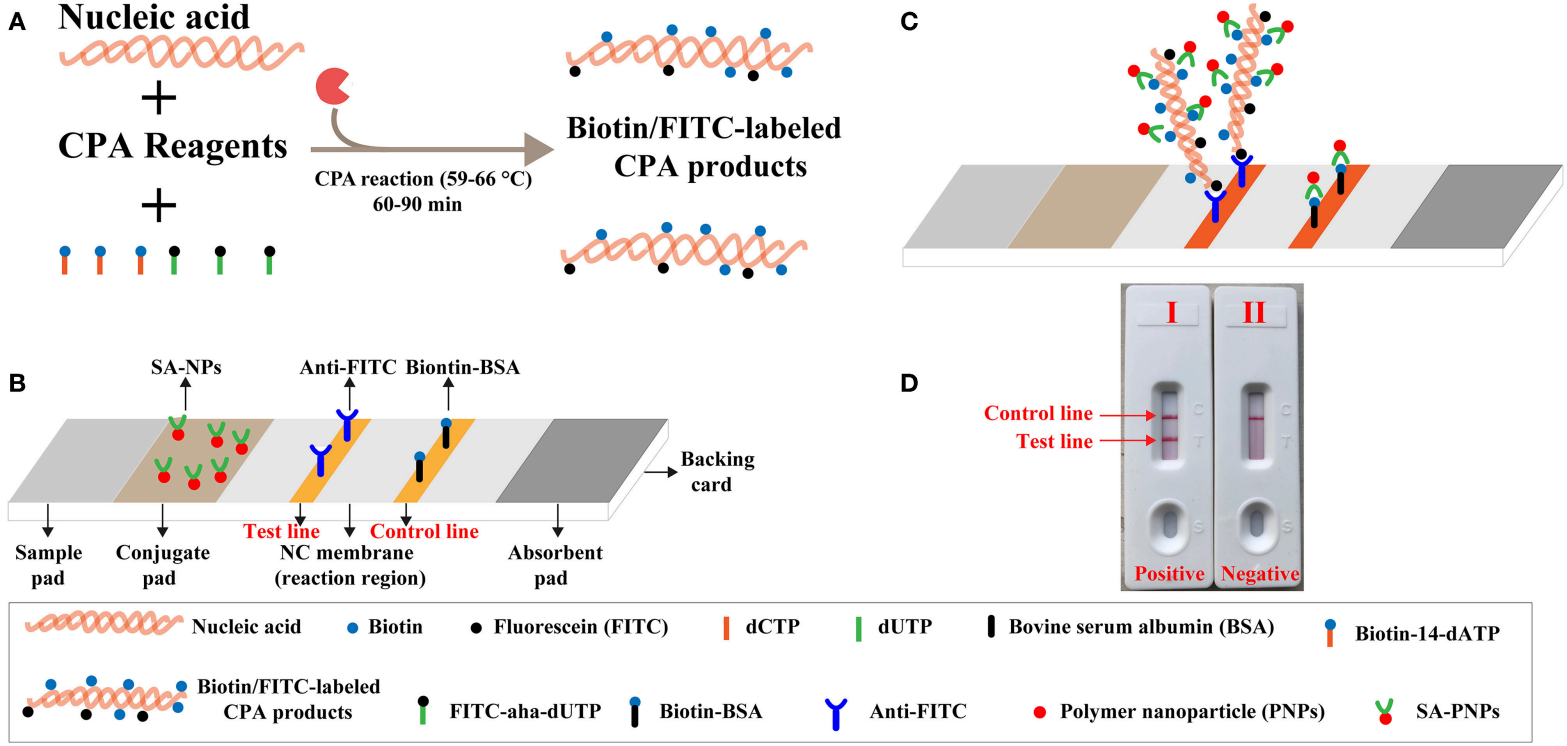
Outline of label-free CPA coupled with lateral flow biosensor (LFB). **(A)** Outline of label-free CPA with biotin-14-dATP and FITC-aha-dUTP. **(B)** The detailed structure of LFB. **(C)** Schematic illustration of the principle of LFB for visualization of CPA products. **(D)** Interpretation of the LFB results: (I), positive (two crimson red bands, including test line and control line, appeared on the NC regions of the biosensor); (II), negative (only the control line zone showed a crimson red line).

To address the shortcomings posed by traditional labeled strategy, a novel strategy for reporting CPA results by biosensor, termed label-free cross-priming amplification coupled with nanoparticles-based lateral flow biosensor (label-free CPA-LFB), was devised at the current study (Figure 1). Label-free CPA-LFB assay eliminates the use of labeled CPA primer or labeled probes, thus the technique effectively removes the false-positive results yielded from interaction between two modified CPA primers. Particularly, opening of CPA reaction tube is an essential step when CPA results are indicated by biosensor or gel electrophoresis. The procedure step easily produces aerosol droplets of different sizes, which contain high concentration of amplification products (Li et al., 2015). Then, these contaminants (aerosol droplets) can become templates for re-amplification, which generated the false-positive results. Toward a strategy for offering the reliable CPA diagnosis and eliminating the carryover contamination, we firstly devised a one-pot, close-vessel enzymatic strategy that is able to effectively prevent carryover contamination while allowing normal CPA amplification and providing accurate identification of target sequences in contaminated samples. Herein, a novel CAP technique (named label-free CPA-ER-LFB), which merged CPA assay with endonuclease restriction (ER) cleavage of contaminants and lateral flow biosensor (LFB) analysis of reaction products, was established for accurate, rapid, sensitive and simple detection of target sequences.

*Staphylococcus aureus* (*S. aureus*), as a commensal bacterium and a major human pathogen, is responsible for a wide range of clinical infections, including bacteremia, infective endocarditis, skin, pleuropulmonary, soft tissue and device-associated infections (Tong et al., 2015). Thus, advanced diagnostic techniques are required for rapid, sensitive and reliable identification of target pathogen to ensure optimal treatment early in the infection. In seeking such detection assay, we investigate the feasibility of CPA-ER-LFB devised in the current report, based on the amplification of the specific heat-stable nuclease (*nuc*) gene. Here, we expounded the basic label-free CPA-ER-LFB mechanism, and demonstrated its suitability by diagnosing the target pathogen using pure cultures and spiked blood samples.

## Materials and Methods

### Reagents and Apparatus

Isothermal amplification kit (Isothermal® Amplification Kit) and visual reagent (Malachite green, MG) was purchased from Bei-Jing HaiTaiZhengYuan. Co., Ltd. (Beijing, China). DNA extraction kits (QIAamp DNA minikits; Qiagen, Hilden, Germany) were obtained from Qiagen (Beijing, China). Absorbent pad, nitrocellulose membrane (NC), conjugate pad, sample pad and backing card were purchased from Jie-Yi Biotechnology. Co., Ltd. (Shanghai, China). Biotinylated bovine serum albumin (biotin-BSA) and rabbit anti-fluorescein antibody (anti-FITC) were obtained from Abcam. Co., Ltd. (Shanghai, China). Dye streptavidin coated polymer nanoparticles (Crimson red, 129 nm) were purchased from Bangs Laboratories, INC. (Indiana, USA). Endonuclease restriction BpuEI was purchased from New England Biolabs, INC. (Beijing, China). Biotin-14-dATP and FITC-aha-dUTP was purchased from Thermo Scientific. Co., Ltd. (Shanghai, China).

### Assemble of the Lateral Flow Biosensor

The lateral flow biosensor (LFB) was made of a plastic backing, the sample pad, the conjugate pad, the NC membranes, and the absorption pad (Figure 1B) (Wang et al., 2017). After spraying the dye streptavidin coated polymer nanoparticles (SA-NPs) and drying at room temperature, the conjugate pad was prepared. The NC membrane was sprayed by anti-FITC antibody (0.25 mg/ml) and biotin-BSA (2.5 mg/ml) to form one test line and one control line with 5 mm interval, respectively, and then dried in the air at room temperature. Then, LFB assembled with the plastic backing orderly pasted by the sample pad, conjugate pad, NC membranes and absorption pad. All assembled cards were cut into 4 mm wide test strip and stored at 4°C for use.

### Design of CPA Primers

A set of five CPA primers (4s, 2s, 3a, 1a, and 5a) was designed using primer software PRIMER PERMIER 5.0, and was listed in Table 1 and Figure S1. Blast analysis was conducted for confirming that all designed primers were specific to *S. aureus*. Particularly, the five primers were examined for hybrids and hairpin structures using the Integrated DNA Technologies design tool (http:www.idtdna.com/site). The CPA primers were synthesized and purified by TsingKe Biotech Co., Ltd (Beijing, China) at HPLC purification grade.

**Table 1:** The primers used in this study.

**Primers name^**a**^**	**Sequences and modifications**	**Length^**b**^**
4s	5′-CGCTACTAGTTGCTTAGTGTT-3′	21 nt
As (1a+2s)	5′-CAGAACCACTTCTATTTACGCCATTGCTTCAAGTCTAAGTAGCTCAG-3′	47 mer
E-As	5′-CAGAACCACTTCTATTTACGCCATTCTTGAGGCTTCAAGTCTAAGTAGCTCAG-3′	53 nt
3a	5′-CAGAACCACTTCTATTTACGCCATT−3′	25 nt
1a	5′-ATCTGTTTGTGATGCATTTG−3′	20 nt
5a	5′-TGCACTATATACTGTTGGA-3′	19 nt

a *As was the core primer in the CPA system, which consisted of the reverse complementary sequence of 1a region and 2s. E-As was the core primer in the CPA-ER assay, which consisted of the reverse complementary sequence of 1a, recognition site (5′-CTTGAG-3′) and 2s. E-As could be recognized by endonuclease restriction BpuEI*.

b *nt, nucleotide; mer, monomeric*.

### Bacterial Strains and Genomic DNA Preparation

A total of 49 bacterial strains, including 2 *S. aureus* reference strains (ATCC 43300 and ATCC 25923), 26 *S. aureus* isolated strains, and 21 non-*S. aureus* strains, were used in this report (Table 2). The genomic templates were extracted using DNA extraction kits (QIAamp DNA minikits, Hilden, Germany) according to the manufacture's instructions. The extracted templates were quantified using ultraviolet spectrophotometer (Nano drop ND-1000, Caliber, Beijing, China) at A260/280. DNA templates of *S. aureus* reference strain (ATCC 43300) were serially diluted (1 ng, 100 pg, 10 pg, 1 pg, 100 fg, 10 fg, and 1 fg per microliter), and were used for the optimization of CPA-based methods.

**Table 2:** Bacterial strains used in this study.

**Bacteria**	**Strain no. (source of strains)^**a**^**	**No. of strains**	**CPA-ER-LFB result^**b**^**
*Staphylococcus. aureus*	ATCC 43300	1	P
	ATCC 25923	1	P
	Isolated strains	26	P
***Non-S. aureus***			
*Staphylococcus epidemidis*	Isolated strain (GZ-CDC)	1	N
*Streptococcus pneumoniae*	ATCC 700674	1	N
*Klebsiella pneumoniae*	ATCC BAA-2146	1	N
*Listeria monocytogenes*	ATCC EGD-e	1	N
*Listeria innocua*	Isolated strains (CP-CDC)	1	N
*Shigella dysenteriae*	Isolated strains (GZ-CDC)	1	N
*Shigella boydii*	Isolated strains (GZ-CDC)	1	N
*Shigella flexneria*	Isolated strains (GZ-CDC)	1	N
*Shigella sonneri*	Isolated strains (GZ-CDC)	1	N
*Salmonella*	Isolated strains (CP-CDC)	1	N
*Enterococcus faecalis*	ATCC35667	1	N
*Enterococcus faecium*	Isolated strains (CP-CDC)	1	N
*Pseudomonas aeruginosa*	Isolated strain (GZ-CDC)	1	N
*Plesiomonas shigelloides*	Isolated strain (GZ-CDC)	1	N
*Aeromonas hydrophila*	Isolated strain (GZ-CDC)	1	N
*Enterobacter cloacae*	Isolated strains (GZ-CDC)	1	N
*Bntorobater sakazakii*	Isolated strains (CP-CDC)	1	N
*Vibrio parahaemolyticus*	Isolated strains (CP-CDC)	1	N
*Acinetobacter baumannii*	Isolated strains (GZ-CDC)	1	N
*Enterotoxigenic E. coli*	Isolated strains (CP-CDC)	1	N
*Campylobacter jejuni*	ATCC 33291	1	N

a *ATCC, American Type Culture Collection; GZ-CDC, Guizhou Center for Disease Control and Prevention. CP-CDC, Changping District Center for Disease Control and Prevention*.

b *P, positive; N, negative. Only templates extracted from S. aureus strains were detected by the CPA-ER-LFB assay, indicating the extremely high selectivity of the assay*.

### Label-Free CPA and Label-Free CPA-ER Reactions

Label-free CPA was performed in a one-step reaction in a 25-μl mixture containing 12.5 μl 2 X of the supplied buffer (Bei-Jing HaitaiZhengYuan), 0.8 μM of 4s primer, 2.4 μM of As primer, 1.2 μM of 3a primer, 1.2 μM of 1a primer and 1.2 μM of 5a primer, 0.5 μl of 50 mM biotin-14-dATP, 0.5 μl of 50 mM FITC-aha-dUTP, 1 μl (8 U) of *Bst* 2.0 DNA polymerase, and 1 μl DNA template.

Label-free CPA-ER (Cross-priming amplification coupled with endonuclease restriction) was also performed in a one-step reaction in a 25-μl mixture containing 12.5 μl 2 X of the supplied buffer (Bei-Jing HaitaiZhengYuan), 0.8 μM of 4s primer, 2.4 μM of As primer, 1.2 μM of 3a primer, 1.2 μM of 1a primer and 1.2 μM of 5a primer, 0.5 μl of 50 mM biotin-14-dATP, 0.5 μl of 50 mM FITC-aha-dUTP, 1 μl (8 U) of *Bst* 2.0 DNA polymerase, 0.5 μl (0.5 U) of endonuclease restriction BpuEI, and 1 μl DNA template.

Three monitoring techniques, including visual reagent (MG), LFB and real-time turbidity (LA-320c), were used for indicating label-free CPA amplification results. Eight reaction temperatures (60–67°C at 1°C interval) were tested for screening the optimal amplification conditions. Amplification mixtures with 1 μl DNA template of *Listera monocytogenes* (*L. monocytogenes*) and *Klebsiella pneumoniae* (*K. pneumoniae*) were selected as negative controls (NC), and amplification mixtures with 1 μl of double distilled water (DW) were used as a blank control (BC).

### Sensitivity of CPA Assays

Limit of detection (LoD) of label-free CPA assays was examined using serial dilutions (1 ng, 100 pg, 10 pg, 1 pg, 100 fg, 10 fg, and 1 fg per microliter) of purified templates of *S. aureus* (ATCC 43300). A volume of 1 μl of each dilution was added into the reaction mixtures, and assay's sensitivity was confirmed as the last dilution of each positive test. All examinations were performed in triplicate.

### Simulating Carryover Contamination

Amplification products, which were produced from label-free CPA-ER reaction at the level of 100 pg μl^−1^, were quantitated using ultraviolet spectrophotometer (NanoDrop ND-1000, Caliber, Beijing, China). Amplification products were serially diluted (10-fold) from 1 × 10^−12^ to 1 × 10^−19^ g μL^−1^, which were used as the DNA templates for label-free CPA and label-free CPA-ER reactions to simulate carryover contamination. One microliter of artificially contaminated templates was added into amplification mixtures.

### Prevention of Carryover Contamination by CPA-ER

To confirm the feasibility of label-free CPA-ER technique to eliminate the unwanted amplification due to carryover contaminants in detecting target templates, we compared label-free CPA method with label-free CPA-ER assay by adding 1 μl of diluted DNA and 1 μl of simulated carryover contamination of 1 × 10^−18^ g μl^−1^ in the same reaction tube. Total mass (1 × 10^−18^ g) of contaminants (label-free CPA-ER amplicons) was equivalent to a 0.2 μm-diameter aerosol droplet, which could not be effectively removed by either fibrous pipette tip filters or high efficiency particulate air filters in the biosafety cabinets. Hence, total mass (1 × 10^−18^ g) of label-free CPA-ER amplicons was employed as the source of simulating carryover contaminants for label-free CPA and label-free CPA-ER amplifications. Sensitivity of label-free CPA and label-free CPA-ER was compared to validate whether the label-free CPA-ER method established here could efficiently preclude false-positive amplifications.

### Specificity of CPA-ER-LFB Assay

To investigate the specificity of label-free CPA-ER-LFB assay, a series of pathogens, including 2 *S. aureus* reference strains, 26 *S. aureus* isolated strains and 21 non-*S. aureus* strains, was tested (Table 2). Each with a concentration of 10 ng was examined after CPA-ER with LFB. Reference strain (*S. aureus* ATCC 43300) was used as a positive strain, and double distilled water (DW) was used as the negative control.

### Evaluation of the Feasibility of Label-Free CPA-ER-LFB Technique

To test the lowest number of bacterial cells detectable by the label-free CPA-ER-LFB technique, serial 10-fold dilutions of *S. aureus* (ATCC 43300) cells were prepared and employed for spiking blood samples. A suspension of ATCC 43300 strain (7 × 10^7^ CFU ml^−1^) was prepared in 1-ml of phosphate-buffered saline, and was applied for making a serial dilution (7 × 10^6^ CFU ml^−1^, 7 × 10^5^ CFU ml^−1^, 7 × 10^4^ CFU ml^−1^, 7 × 10^3^ CFU ml^−1^, 7 × 10^2^ CFU ml^−1^, 7 × 10^1^ CFU ml^−1^, and 7 × 10^0^ CFU ml^−1^). Each dilution was centrifuged, which was re-suspended in 100 μl of blood sample (a 5-day negative blood culture). The artificially blood samples was subjected to extracted genomic DNA, and the templates were eluted in 100 μl of elution buffer. A volume of 2 μl of extracted DNA was employed for conducting label-free CPA-ER-LFB reactions. Non-contamination samples were employed as the negative control. All examinations were performed in duplicate to ensure accuracy and reproducibility.

## Results

### The Development of Label-Free CPA-LFB Assay

A schematic illustration of the mechanism of label-free CPA-LFB was displayed in Figure 1. In the label-free CPA-LFB analysis system, two components, including biotin-14-dATP and FITC-aha-dUTP, were simultaneously added into the amplification mixtures (Figure 1A). CPA primers anneal to target templates during the amplification stage, and they were extended by *Bst* 2.0 polymerase. Thus, the two components (Biotin-14-dATP and FITC-aha-dUTP) could be incorporated into the CPA products. As a result, all CPA complicons were simultaneously modified with biotin and FITC. The biotin- and FITC-labeled CPA products were termed as double-labeled detectable amplicons, which could be visually detected by LFB.

A schematic illustration of the mechanism of LFB for visual analysis of label-free CPA products was exhibited in Figure 1C. A volume of 2 μl of label-free CPA amplification mixtures was loaded into the sample region, and a volume of 60 μl of diluent buffer also was deposited on sample region. Diluent buffer rehydrated the fixed detector reagents (SA-PNPs) at the conjugate pad, because diluent buffer could move along the LFB by capillary action. Double-labeled detectable CPA amplicons were specifically recognized at the test line (TL) through anti-FITC body capture of FITC labels. Then, SA-PNPs (detector reagent) could accumulate on the test zone through biotin/streptavidin interaction (Figures 1B,C), which produced a visual crimson red band in the TL region of LFB. Furthermore, excess SA-PNPs (detector reagents) could be captured by immobilized biotinylated bovine serum albumin (biotin-BSA) on the control line (CL), which validated the proper function of LFB (Figures 1C,D).

### Confirmation of CPA Products

To examine the feasibility of CPA primer set devised in this study, label-free CPA amplifications were conducted in the presence or absence of templates within 1-h at a fixed temperature (63°C). The amplified products of the positive reaction were visualized with MG reagents, and the color of the amplification mixtures changed from colorless to light green (Figure S2A). However, the color of solution remained colorless in the negative reactions and blank control (Figure S2A). Using LFB, two crimson red bands (TL and CL) were observed in the positive CPA amplifications, and only a crimson red band (CL) was seen in the negative controls and blank control (Figure S2B). These data suggested that the CPA primer set designed in this report was a suitable candidate for establishing the label-free CPA-ER-LFB technique.

### Optimal Amplification Temperature of Label-Free CPA Assay

The optimal temperature for label-free CPA reaction were determined with 1 pg of *S. aureus* ATCC 43300 templates under isothermal conditions at temperature of 60°C to 67°C for 1-h by monitoring turbidity. Although amplification targeting of the *nuc* gene was detected at all of the temperatures examined, a threshold value of absorbance (0.1) from the label-free CPA method was reached most quickly at 63°C (Figure S3). No non-specific amplification was seen in the negative controls and blank control after 1-h of incubation. Therefore, the subsequent label-free CPA reactions were carried out at 63°C for 1-h.

### Sensitivity of CPA-LFB Assay in Pure Culture

**Figure 2:**
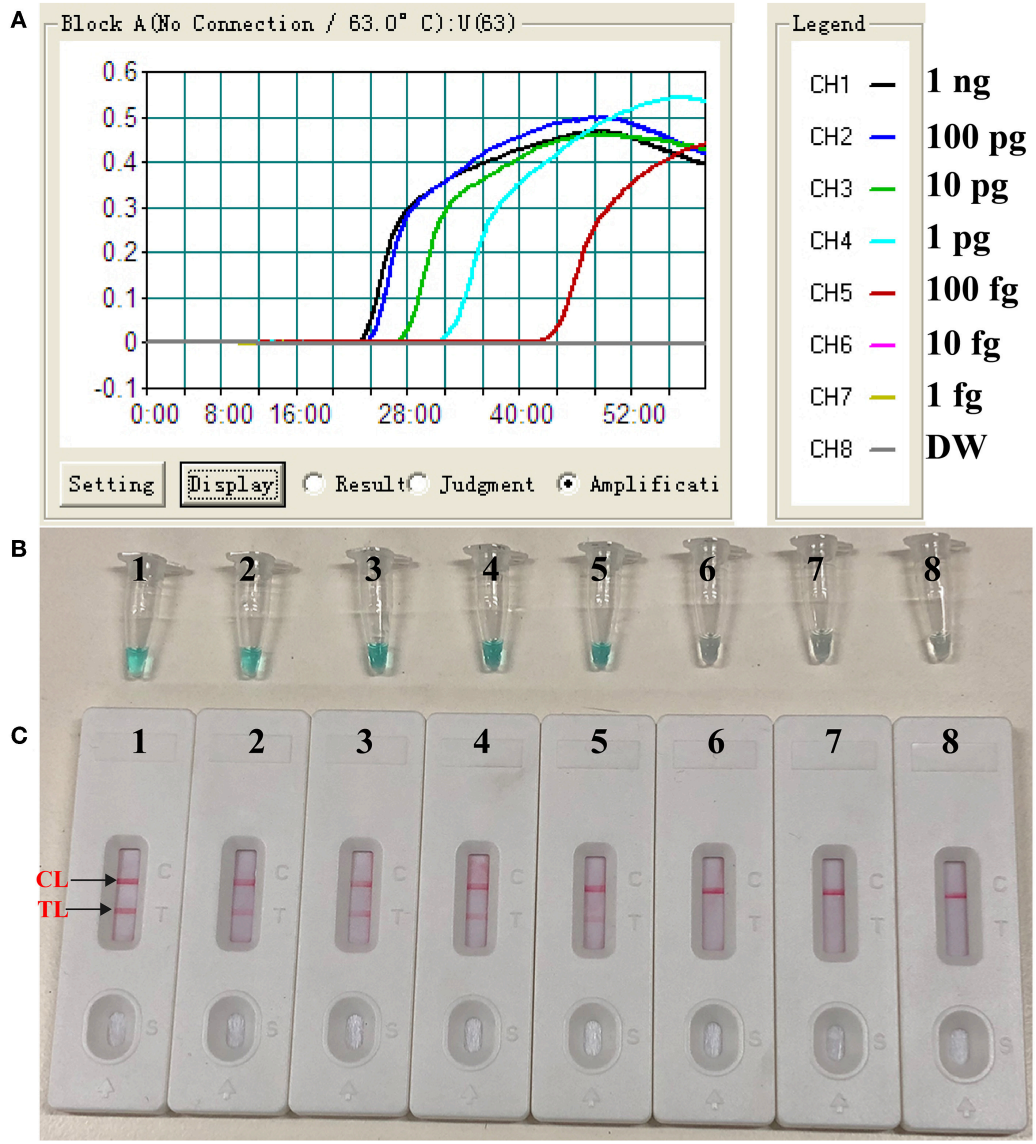
Sensitivity of label-free CPA-LFB assay using serially diluted *S. aureus* (ATCC 43300) templates. Real-time turbidity **(A)**, Colorimetric indicator (MG, **B**) and biosensor **(C)** were applied for analyzing the CPA reactions products. Signals/Tubes/biosensors 1, 2, 3, 4, 5, 6, and 7 represent the genomic DNA levels of 1 ng.μL^−1^, 100 pg.μL^−1^, 10 pg.μL^−1^, 1 pg.μL^−1^, 100 fg.μL^−1^, 10 fg.μL^−1^, and 1 fg.μL^−1^. Tube/biosensor 8, blank control (DW).

To evaluate the sensitivity of the label-free CPA method, the target assay was performed using equivalent quantities of genomic DNA extracted from pure culture sample (*S. aureus* ATCC 43300) as templates at various dilutions (1 ng, 100 pg, 10 pg, 1 pg, 100 fg, 10 fg, and 1 fg per microliter). The label-free CPA amplified the targeted sequence at each dilution from the highest concentration (1 ng per microliter) to as little as 100 fg of genomic DNA per reaction (Figure 2). Using LFB, two visual lines (TL and CL) appeared in the positive results, and only a visual band was observed in the negative results (Figure 2C). Using the label-free CPA amplification by self-trail, sensitivity of label-free CPA was further validated by turbidity analysis and direct visual inspection of reactions products with MG reagents (Figures 2A,B). Sensitivity of label-free CPA assay obtained using turbidity analysis and MG reagents was also 100 fg of DNA per tube, which was in complete accordance with LFB detection (Figure 5).

### CPA-ER Assay

**Figure 3:**
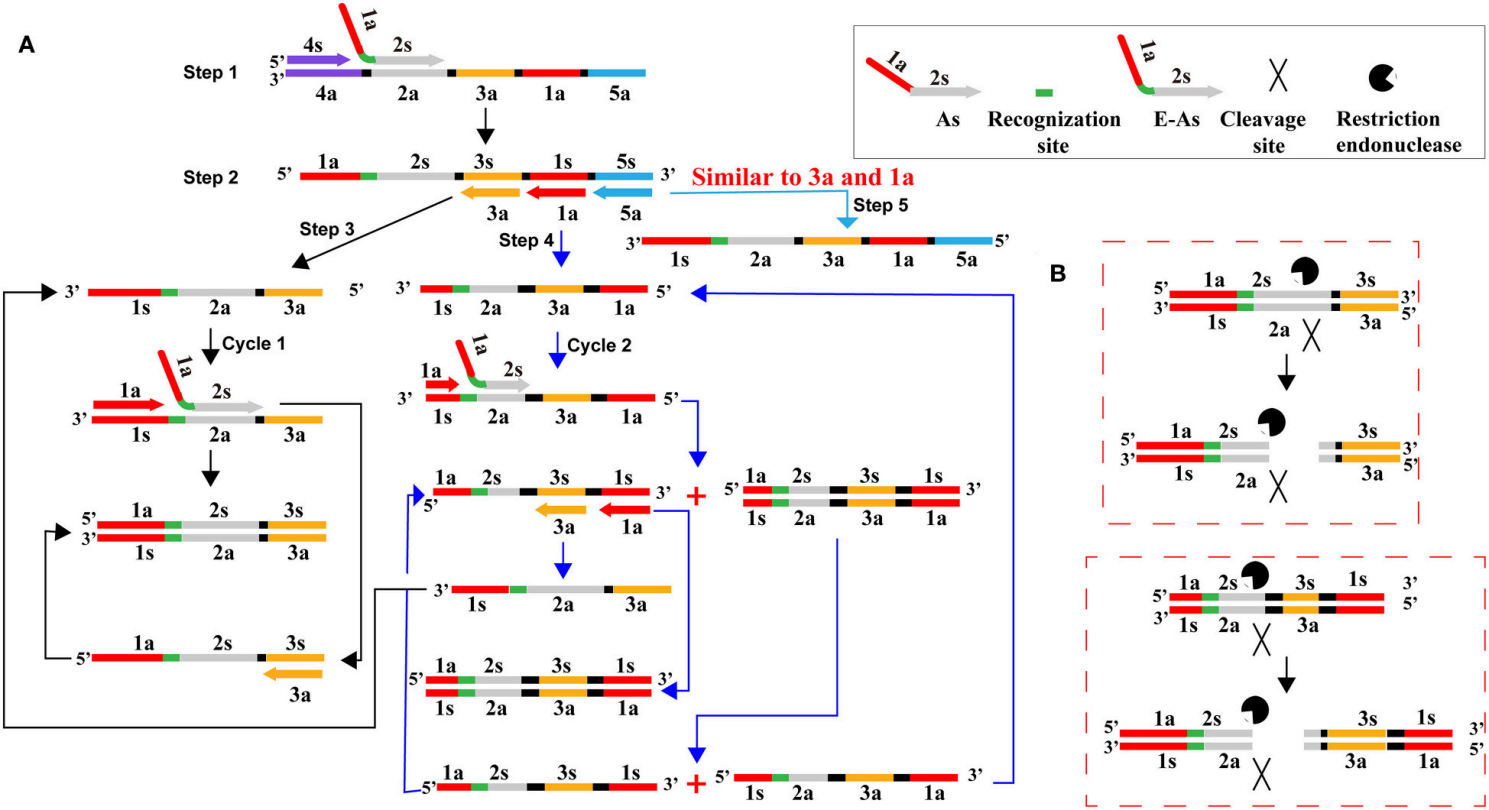
Schematic illustration of CPA-ER pollution elimination by a primer enzymatic method. **(A)** CPA amplification with the core primer (As) carrying the recognition site of endonuclease restriction BpuEI. **(B)** Schematic presentation of digesting carryover CPA amplicons. CPA amplification products were cut into several kinds of DNA strands by endonuclease restriction BpuEI. As was the core primer in the CPA system, which consisted of the reverse complementary sequence of 1a region and 2s. E-As was the core primer in the CPA-ER assay, which consisted of the reverse complementary sequence of 1a, recognition site, and 2s.

In this report, we devised a primer enzymatic strategy to remove carryover contaminants of previous CPA reactions, which was designated as CPA-ER (Cross-priming amplification combined endonuclease restriction) method (Figure 3). The As primer (core primer) of CPA assay was designed to carry the recognition site (RS) of restriction endonuclease BpuEI, and the new As primer was termed as E-As (Figure 3A). RS was inserted between regions 1a and 2s in AS primer. The CPA-ER reaction (CPA coupled with endonuclease restriction, CPA-ER) was performed with the engineered As primer (E-As), and the final CPA products of which were a mixture of DNAs including the RS of endonuclease restriction (BpuEI). In order to prevent carryover contamination of CPA, prior to next CPA reaction, we treated the CPA amplification mixtures with BpuEI to cleave carryover contaminations of previous CPA amplifications. The carryover CPA amplicons were recognized at RS, and cut at 2s by endonuclease restriction BpuEI. Thus, the engineered core primer (E-As) did not anneal with carryover amplicons of CPA, effectively eliminating carryover contamination from re-amplification. Particularly, the recognition site and cutting site of endonuclease restriction should have an appropriate gap to ensure the post-cut carryover amplicons not to stably hybridize with core primer E-As. BpuEI was an endonuclease restriction that was able to cleave at 16th base behind the recognition site, thus the bases of the post-cut carryover reaction products were extremely difficult to stably anneal with primers. In contrast, the natural genomic templates and the CPA primer molecules did not be affected in the process of pretreatment. Significantly, CPA-ER assay could be conducted in a closed-tube because BpuEI was heat-labile to be deactivated at 65°C, but *Bst* 2.0 DNA polymerase was activated, permitting CPA amplification reaction to normally proceed. As a result, reaction products from CPA-ER amplification could be analyzed by colorimetric indictor, real-time turbidity and lateral flow biosensor.

### CPA-ER Method Detects Simulated Carryover Contamination

**Figure 4:**
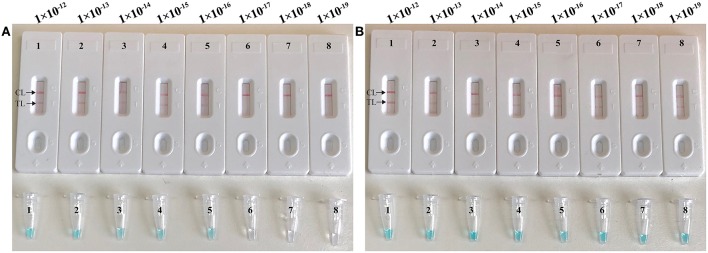
Control of carryover contamination in CPA-ER assay. Sensitivity of conventional CPA-ER **(A)** and CPA **(B)** using serial dilution of simulated carryover contamination (1 × 10^−12^, 1 × 10^−13^, 1 × 10^−14^, 1 × 10^−15^, 1 × 10^−16^, 1 × 10^−17^, 1 × 10^−18^, and 1 × 10^−19^ g.μL^−1^) as determined using colorimetric indicator (Top row) and biosensor (Bottom row).

To validate that simulated carryover contaminants from previous CPA amplicons is capability of contaminating new CPA amplification system. We performed CPA-ER and standard CPA reactions using serial diluted CPA-ER amplicons with concentrations ranging from 1 × 10^−12^ to 1 × 10^−19^ g/μL (Figure 4). The CPA-ER method could detect 1 × 10^−16^ g of contaminants per vessel (Figure 4A), and standard CPA detect as little as 1 × 10^−19^ g of contaminants per tube (Figure 4B). Our data confirmed that marginal amount of CPA amplicons (1 × 10^−18^ g~0.2 μm-diameter aerosol droplet), which were not be removed by fibrous pipette tip filters, could produce unwanted amplifications. Thus, the CPA-ER assay was able to eliminate CPA amplicons up to 10^3^-fold higher concentrations of contaminants (1 × 10^−19^ g), which significantly reduce the likelihood of unwanted results of CPA assay.

### CPA-ER Eliminates False-Positive Results

**Figure 5:**
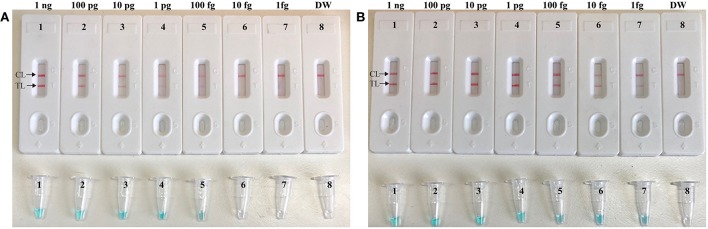
CPA-ER method prevent false-positive result due to carryover contamination. Sensitivity of conventional CPA-ER **(A)** and CPA technique **(B)** using serial dilutions (1 ng.μl^−1^, 100 pg.μl^−1^, 10 pg.μl^−1^, 1 pg.μl^−1^, 100 fg.μl^−1^, 10 fg.μl^−1^, and 1 fg.μl^−1^) of ATCC 43300 and 1 × 10^−18^ g.μL^−1^ of simulated carryover contamination as determined using LFB (Top row) and colorimetric indicator (Bottom row).

To further confirmed that CPA-ER method could abate the likelihood of unwanted results due to contaminants, the sensitivity of CPA-ER and standard CPA assays were conducted using serial dilution of the *S. aureus* (ATCC 43300). Simultaneously, CPA-ER products at the level of 1 × 10^−18^ g, which were used as the source of carryover contamination, also are added into CPA-ER and CPA reactions. All determined samples displayed positive reactions in the standard CPA method, even containing these samples with undetectable level of target templates (< 100 fg per tube), which were named as false-positive amplifications (Figure 5B). In contrast, the sensitivity of CPA-ER method was complete accordance with the aforementioned sensitivity evaluations (Figures 2, 5A). These data suggested that standard CPA technique could not correctly verify the approach's sensitivity, and CPA-ER technique established here could effectively eliminate unwanted results produced from carryover contamination.

### Specificity of Label-Free CPA-ER-LFB Assay

The specificity of CPA-ER-LFB assay was determined by examining 2 *S. aureus* reference strains, 26 *S. aureus* isolated strains and 21 non-*S. aureus* strains. The results were positive for all *S. aureus* strains, and negative for other types of non-*S. aureus* strains (Table 2). These date demonstrated that the label-free CPA-ER-LFB method detected all known *S. aureus* strains.

### Label-Free CPA-ER-LFB Assay to *S. aureus* in Spiking Blood Samples

**Figure 6:**
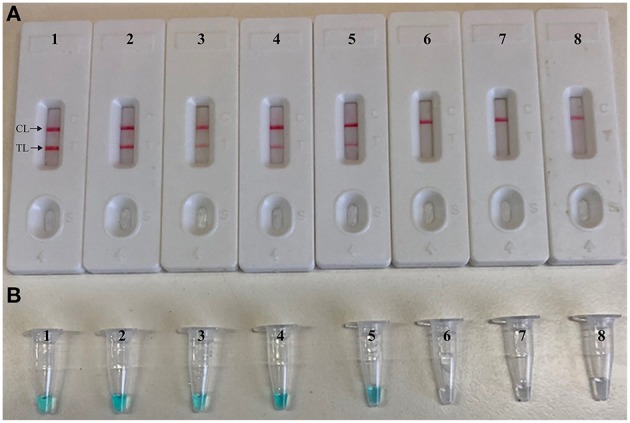
Sensitivity of label-free CPA-ER-LFB for detecting *S. aureus* in blood samples. Monitoring techniques, including biosensor **(A)** and colorimetric indicator (MG, **B**), were applied for reporting label-free CPA-ER-LFB results. Ten-fold serial dilutions of target bacterium were subjected to label-free CPA-ER-LFB reaction. Biosensor **(A)**/Tubes **(B)** 1–8 represented the cell levels of 140,000 CFU, 14,000 CFU, 1,400 CFU, 140 CFU, 14 CFU, 1.4 CFU, 0.14 CFU per reaction, negative control (non-contaminated sputum samples). The cell levels of 140,000 CFU, 14,000 CFU, 1,400 CFU, 140 CFU, and 14 CFU per reaction yielded the positive amplifications.

To further validate the feasibility of label-free CPA-ER-LFB as a valuable tool for target pathogen detection, we determine the artificially contaminated blood samples with *S. aureus* strain (ATCC 43300) by using label-free CPA-ER-LFB method. The label-free CPA-ER-LFB assay produced the positive signals when the contaminated numbers of *S. aureus* (ATCC 43300) were more than 7 × 10^3^ CFU ml^−1^ (14 CFU per tube) (Figure 6). The label-free CPA-ER-LFB generated the negative results when the contaminated numbers of *S. aureus* (ATCC 4330) were less than 7 × 10^2^ CFU ml^−1^ (1.4 CFU per reaction). Furthermore, we did not observe the positive signals in negative controls (non-spiked blood samples).

## Discussion

**C**ross priming amplification (CPA), as an isothermal exponential amplification technique, has great potential for point-of-care, on-site, field and *in-situ* assay applications (Huo et al., 2017; Feng et al., 2018b). Up to now, this amplification assay has been employed to detect a variety of pathogens (such as bacteria, viruses, parasites, and fungi). However, the wider application of CPA was hindered because of the difficulties of visualization, complexity of result analysis. Traditionally, monitoring techniques, including agarose gel electrophoresis, fluorescent agents, colorimetric agents and turbidimeters, have been used for indicating CPA results (Feng et al., 2018b). Unfortunately, these monitoring techniques were not able to differentiate specific amplification products and non-specific amplification products. In addition, traditional analysis methods required an additional detection procedure (gel electrophoresis), special reagents (such as fluorescent) or complicated apparatus (such as turbidimeters), which further prevented CPA method from being widely used in various fields (Gou et al., 2018).

These disadvantages shared by traditional monitoring techniques have spurred the researchers to devise other superior analysis technique. Accordingly, lateral flow biosensor (LFB) has been developed for solving such shortcomings above, due to the excellent traits of visual results interpretation, pollution-free, user-friendly, reliable, and rapid signal output in low-resource settings (Qiao et al., 2016; Huo et al., 2017; Gou et al., 2018; Meng et al., 2018). In this report, we established a novel label-free CPA-LFB method to construct the double-labeled detectable CPA amplicons, which could be detected by LFB. In the label-free CPA-LFB system, the detectable CPA products were formed from biotin-14-dATP and FITC-aha-dUTP during the amplification stage, eliminating the use of hapten-labeled primers or hapten-labeled probes. The FITC tag, which was incorporated into amplicons using FITC-aha-dUTP, was specifically captured by the anti-FITC body immobilized at the test region of the biosensor (Figures 1B,C). The biotin tag, which was incorporated into amplicons using biotin-14-dATP, was capability of binding dye streptavidin coated polymer nanoparticles (SA-PNAs) for visualization. The CPA results were indicated as a crimson red band visible by the naked eye within 5-min (Figures 1C,D). Comparing with traditional monitoring methods, LFB displayed the advantages on easy operation, cost efficiency, disposable format, visual, and quick result. Due to eliminate the use of the labeled-primers or labeled-probes, label-free CPA-LFB assay developed in the current report could remove undesired results arising from the hybridization between two labeled primers or between a labeled primer and probe. In addition, the LFB devised in the study could directly analyzed the target amplicons without heating inactivation and hybridization steps, which were essential processes for previously established LFB protocol (Yulong et al., 2010; Prompamorn et al., 2011).

CPA technique, as an isothermal amplification method with high sensitivity, has been applied in many fields. Just because of its excellent sensitivity, the unwanted results has been puzzling researchers for a long time due to re-amplification from previous CPA amplicons, namely carryover contamination. CPA products with high concentration might easily form aerosols or splash during experimental operation (such as reporting the CPA results by agarose gel electrophoresis or LFB), because the opening of reaction vessel was essential step. Thus, these procedures could cause pollution to reagents, air, pipettes, the clothes and gloves of experimenter. The results suggested that trace amounts of CPA amplicons (1 × 10^−19^ g/reaction) were able to produce false-positive amplifications (Figure 4). Hence, preventing carryover contamination was an excellently important factor to achieve reliable and accurate CPA detection. To remove the carryover contamination in CPA reaction, we devised a primer enzymatic technique to prevent carryover contaminants of previous CPA amplifications, termed as CPA-ER assay. In the CPA-ER system, CPA core primer was designed to carry the recognition site of restriction endonuclease between regions 1a and 2s (Table 1 and Figure 3), thus the final CPA amplicons of which were a mixture of products containing the recognition site of endonuclease restriction. Then, endonuclease restriction digestion and isothermal reaction were performed in a one-pot process. Prior to next isothermal amplification, the CPA reaction mixtures were treated with endonuclease restriction to digest the carryover contaminations, which were cut at 2s region (Figure 3). Therefore, the engineered core primer did not anneal with carryover amplicons of CPA, effectively preventing carryover contamination from re-amplification. However, the target templates and the primer molecules could not be cut in the process of pretreatment, because the natural DNA did not contain the recognition site and the endonuclease site was not able to cleave the single-strand primer molecules. Importantly, CPA-ER amplification could be carried out in a closed-tube because endonuclease restriction BpuEI was heat-labile to be deactivated at 65°C for 10 min, but *Bst* 2.0 DNA polymerase was activated, As a result, genuine CPA products subsequently generated from the target templates were not digested during the amplification stage, allowing CPA amplification to proceed normally.

*S. aureus*, as a major human pathogen, was responsible for a series of clinical infections, such as bacteremia, endocarditis, superficial, deep-skin, and soft-tissue infections (Klein et al., 2019). Thus, the rapid detection of this pathogen was excellently important so that the appropriate antibiotic therapy could be initiated. As a poof-of-concept, *S. aureus* was selected as model for demonstrating the feasibility of label-free CPA-ER-LFB assay. A set of five primers was designed for detection of *S. aureus* on the basis of specific *nuc* gene (Figure S1) (Wang et al., 2018b), and the suitability of primer set then was successfully validated by performing the standard CPA reactions in the presence or absence of target templates at a fixed temperature (Figure S2). To optimize the isothermal amplification conditions, a total of ten distinct reaction temperatures (59–68°C, 1°C interval) were tested, and the better result were produced from the assay temperature of 63°C (Figure S3). Our data indicated that the sensitivity of label-free CPA-LFB for *S. aureus* detection was 100 fg of genomic templates per tube in pure culture, which was consistent with label-free CPA-ER-LFB assay (Figures 2, 5). In particular, label-free CPA-ER-LFB assay was capability of removing the carryover contaminations, thus the unwanted results arising from previous CPA products could be efficiently prevented. Moreover, comparing with other monitoring methods (such as agarose gel electrophoresis, real-time turbidity, colorimetric indicator), LFB was likely the preferred analysis technique as indicating the results using biosensor is less subjective and does not rely on special agents or apparatus. In addition, all *S. aureus* strains determined in the current study were positive for label-free CPA-ER-LFB detection, and no positive results were observed in the assay of non-*S. aureus* strains, indicating the high selectivity of the CPA-ER-LFB assay (Table 2). Practical application of label-free CPA-ER-LFB assay was also successfully evaluated by detecting *S. aureus* in spiked blood samples with highly specificity and sensitivity (Figure 6).

## Conclusion

In sum, we devised a new method on the basis of standard CPA assay, termed label-free CPA-ER-LFB. A new reaction system firstly was established to form the detectable CPA products, which did not require the use of labeled primers or labeled probes, and were able to be detected using lateral flow biosensor. Thus, the new strategy was capable of effectively removing the undesired results arising from non-specific hybridization (between two labeled primers or between the labeled primer and probe). Then, our report integrated label-free CPA reaction with endonuclease restriction digestion cleavage in a one-pot, closed-vessel reaction to prevent false-positives generating from carryover contaminants. For demonstration purpose, *S. aureus* was detected by CPA-ER-LFB to validate the usability of target analysis. The sensitivity, reliability and practical application of label-free CPA-ER-LFB assay in diagnosing target bacterium were successfully examined using pure culture and spiked blood samples. As the poof-of-concept method, label-free CPA-ER-LFB assay could be reconfigured to diagnose various targets by redesigning the CPA primer set.

## Author Contributions

YiW conceived and designed the experiments. YiW, LS, JL, ZW, WJ, JX, CS, FX, HQ, YoW, YG, and AS performed the experiments. YiW, LS, JL, ZW, and WJ analyzed the data. YiW, LS, JL, ZW, WJ, JX, CS, FX, HQ, YoW, YG, and AS contributed the reagents, materials, analysis tools. YiW performed the software. YiW and AS wrote the paper.

### Conflict of Interest Statement

The authors declare that the research was conducted in the absence of any commercial or financial relationships that could be construed as a potential conflict of interest.
