# Supplementary material for: Label-Free Cross-Priming Amplification Coupled With Endonuclease Restriction and Nanoparticles-Based Biosensor for Simultaneous Detection of Nucleic Acids and Prevention of Carryover Contamination
Source: Front Chem. 2019 May 8;7:322. doi: 10.3389/fchem.2019.00322 (PMC6517798; doi:10.3389/fchem.2019.00322)
Supplement: Supplementary file 1 [file Table_1.DOC]

**Supplementary**

**Figure S1. Nucleotide sequence of the *nuc* gene used to design the CPA primer set**

The nucleotide sequence of sense strand of *nuc* was listed. The sequences of the primer sites were underlined, right and left arrows indicated sense and complementary sequences that are used.

**Figure S2**. **Confirmation and detection of label free CPA products**

**A**, Colorimetric indicator (MG) applied for visual determination of label free CPA amplification products. **B**, The biosensor applied for visual detection of label free CPA products. Tube/biosensor 1, positive amplification of label free CPA method (*S. aureus*, ATCC 43300); Tube/biosensor 2, negative amplification of label free CPA method (*L. monocytogenes*); Tube/biosensor 3, negative amplification of CPA assay (*K. pneumoniae*); Tube/biosensor 4, blank control (DW).

**Figure S3. Optimal reaction temperature for label free CPA primer set**

Label free CPA reactions for detection of *S. aureus* were monitored by real-time measurement of turbidity (LA-320c). The threshold value was 0.1 and the turbidity of >0.1 was considered as positive reaction. Eight kinetic graphs were obtained at different temperatures (60°C-67°C, 1°C intervals) with target templates at the level of 1 pg per tube. Signal 1, positive amplification of CPA method (*S. aureus*, ATCC43300); Signal 2, negative amplification of CPA method (*L. monocytogenes*); Signal 3, negative amplification of CPA assay (*K. pneumoniae*); Signal 4, blank control (DW).
