# Supplementary figures and images for: Label-Free Cross-Priming Amplification Coupled With Endonuclease Restriction and Nanoparticles-Based Biosensor for Simultaneous Detection of Nucleic Acids and Prevention of Carryover Contamination
Source: Front Chem. 2019 May 8;7:322. doi: 10.3389/fchem.2019.00322 (PMC6517798; doi:10.3389/fchem.2019.00322)

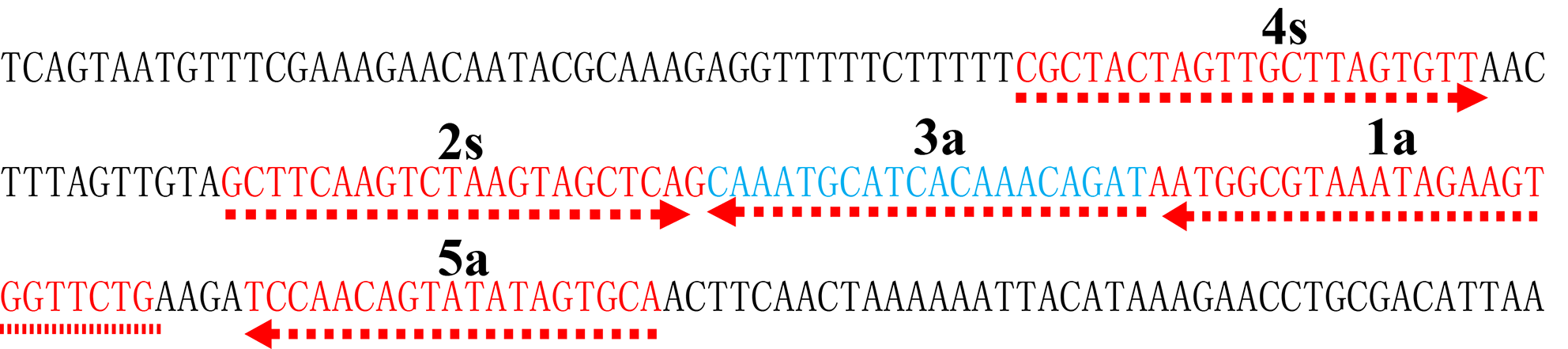

Supplement: Supplementary file 2 [file Image_1.TIF]

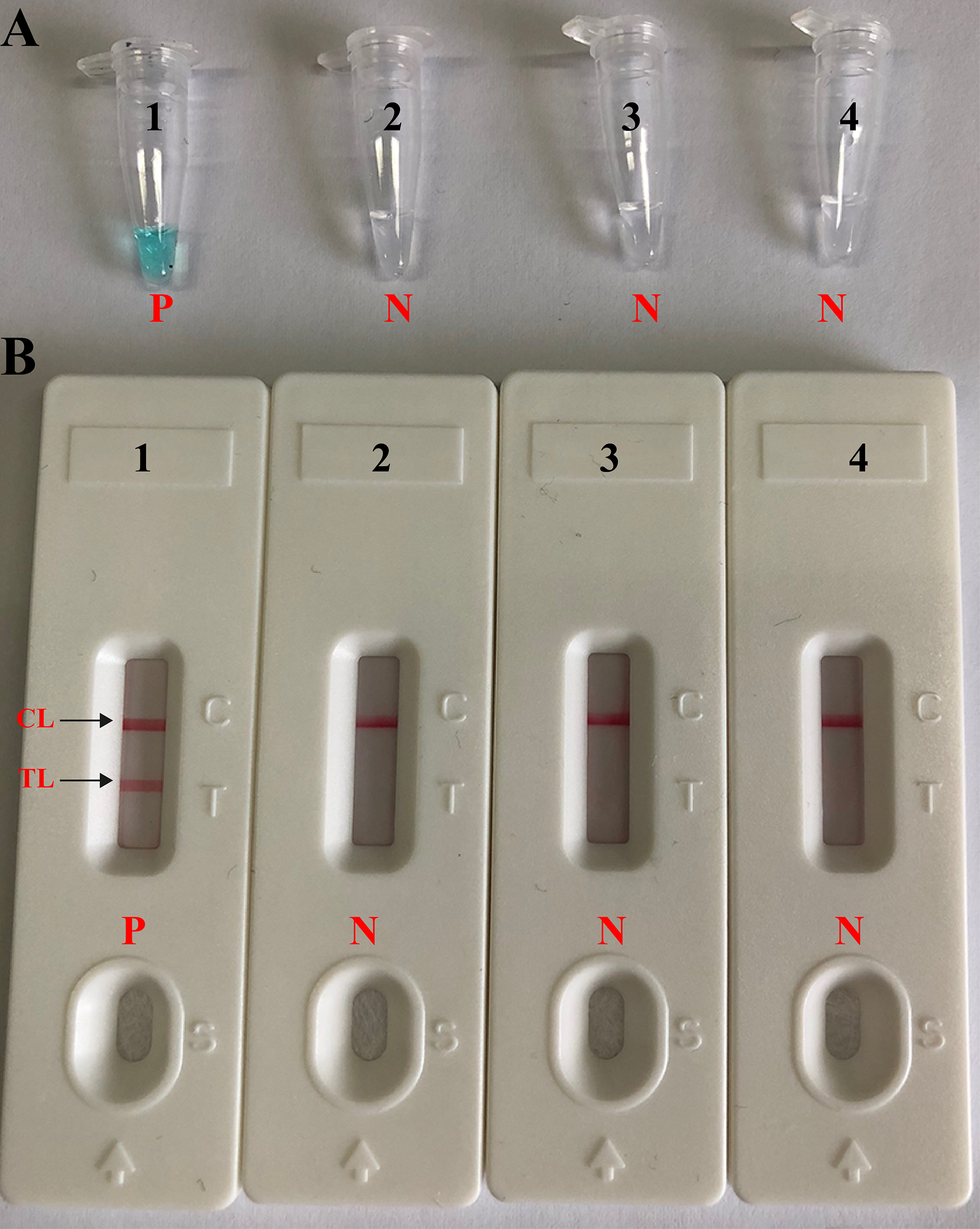

Supplement: Supplementary file 3 [file Image_2.TIF]

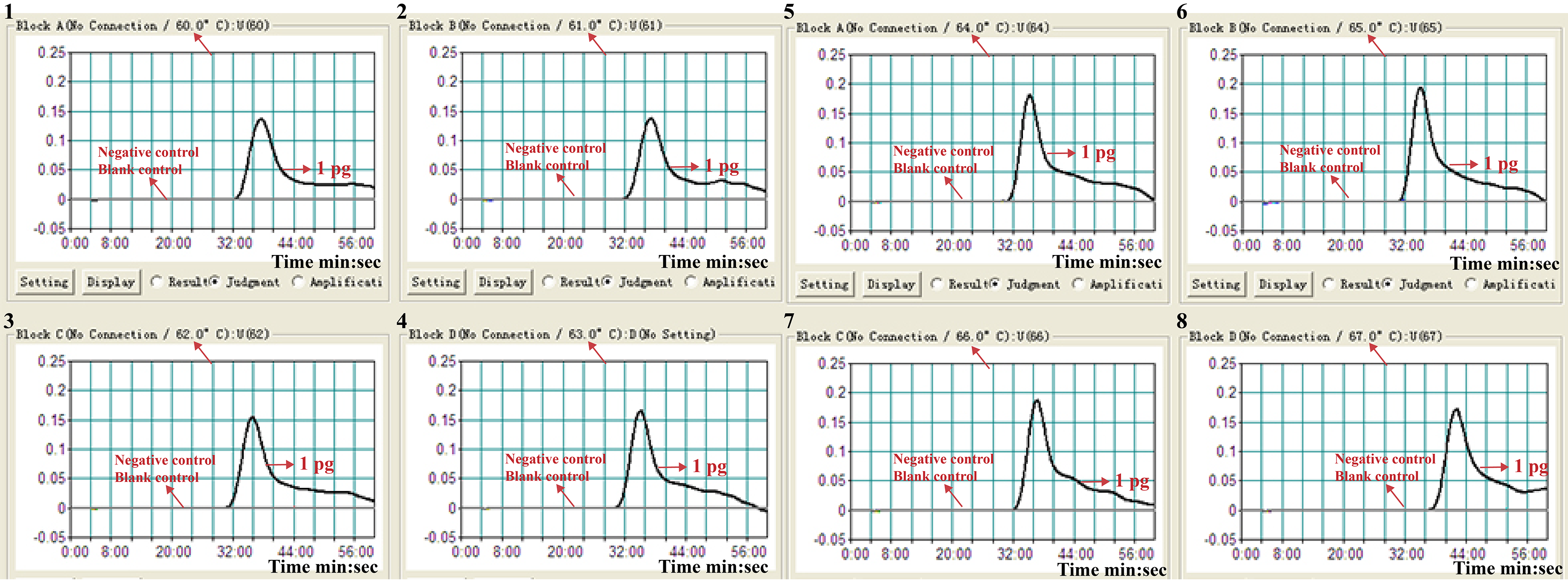

Supplement: Supplementary file 4 [file Image_3.TIF]
